# PAX4 preserves endoplasmic reticulum integrity preventing beta cell degeneration in a mouse model of type 1 diabetes mellitus

**DOI:** 10.1007/s00125-016-3864-0

**Published:** 2016-01-26

**Authors:** José Manuel Mellado-Gil, Carmen María Jiménez-Moreno, Alejandro Martin-Montalvo, Ana Isabel Alvarez-Mercado, Esther Fuente-Martin, Nadia Cobo-Vuilleumier, Petra Isabel Lorenzo, Eva Bru-Tari, Irene de Gracia Herrera-Gómez, Livia López-Noriega, Javier Pérez-Florido, Javier Santoyo-López, Andreas Spyrantis, Paolo Meda, Bernhard O. Boehm, Ivan Quesada, Benoit R. Gauthier

**Affiliations:** Pancreatic Islet Development and Regeneration Unit, Department of Stem Cells, Andalusian Center for Molecular Biology and Regenerative Medicine (CABIMER), Avda Américo Vespucio, Parque Científico y Tecnológico Cartuja 93, 41092 Seville, Spain; Centro de Investigación Biomédica en Red de Diabetes y Enfermedades Metabólicas Asociadas (CIBERDEM), Spain, http://www.ciberdem.org; Instituto de Bioingeniería, Universidad Miguel Hernandez, Elche, Spain; Medical Genome Project, Genomics & Bioinformatics Platform of Andalusia, Seville, Spain; Edinburgh Genomics, University of Edinburgh, Edinburgh, UK; Department of Internal Medicine, Ulm University Medical Centre, Ulm, Germany; Department of Cell Physiology and Metabolism, University of Geneva, Geneva, Switzerland; Lee Kong Chian School of Medicine, Nanyang Technological University, Singapore, Republic of Singapore; Imperial College, London, UK

**Keywords:** Beta cell degeneration, ER homeostasis, PAX4, RIP-B7.1, Type 1 diabetes

## Abstract

**Aims/hypothesis:**

A strategy to enhance pancreatic islet functional beta cell mass (BCM) while restraining inflammation, through the manipulation of molecular and cellular targets, would provide a means to counteract the deteriorating glycaemic control associated with diabetes mellitus. The aims of the current study were to investigate the therapeutic potential of such a target, the islet-enriched and diabetes-linked transcription factor paired box 4 (PAX4), to restrain experimental autoimmune diabetes (EAD) in the RIP-B7.1 mouse model background and to characterise putative cellular mechanisms associated with preserved BCM.

**Methods:**

Two groups of RIP-B7.1 mice were genetically engineered to: (1) conditionally express either PAX4 (BPTL) or its diabetes-linked mutant variant R129W (mutBPTL) using doxycycline (DOX); and (2) constitutively express luciferase in beta cells through the use of RIP. Mice were treated or not with DOX, and EAD was induced by immunisation with a murine preproinsulin II cDNA expression plasmid. The development of hyperglycaemia was monitored for up to 4 weeks following immunisation and alterations in the BCM were assessed weekly by non-invasive in vivo bioluminescence intensity (BLI). In parallel, BCM, islet cell proliferation and apoptosis were evaluated by immunocytochemistry. Alterations in PAX4- and PAX4R129W-mediated islet gene expression were investigated by microarray profiling. PAX4 preservation of endoplasmic reticulum (ER) homeostasis was assessed using thapsigargin, electron microscopy and intracellular calcium measurements.

**Results:**

PAX4 overexpression blunted EAD, whereas the diabetes-linked mutant variant PAX4R129W did not convey protection. PAX4-expressing islets exhibited reduced insulitis and decreased beta cell apoptosis, correlating with diminished DNA damage and increased islet cell proliferation. Microarray profiling revealed that PAX4 but not PAX4R129W targeted expression of genes implicated in cell cycle and ER homeostasis. Consistent with the latter, islets overexpressing PAX4 were protected against thapsigargin-mediated ER-stress-related apoptosis. Luminal swelling associated with ER stress induced by thapsigargin was rescued in PAX4-overexpressing beta cells, correlating with preserved cytosolic calcium oscillations in response to glucose. In contrast, RNA interference mediated repression of PAX4-sensitised MIN6 cells to thapsigargin cell death.

**Conclusions/interpretation:**

The coordinated regulation of distinct cellular pathways particularly related to ER homeostasis by PAX4 not achieved by the mutant variant PAX4R129W alleviates beta cell degeneration and protects against diabetes mellitus. The raw data for the RNA microarray described herein are accessible in the Gene Expression Omnibus database under accession number GSE62846.

**Electronic supplementary material:**

The online version of this article (doi:10.1007/s00125-016-3864-0) contains peer-reviewed but unedited supplementary material, which is available to authorised users.

## Introduction

The islet of Langerhans is the core unit of the endocrine pancreas, which regulates blood glucose homeostasis. Regulation is achieved by the release of insulin from beta cells in response to increasing levels of glucose and by the secretion of glucagon from alpha cells under fasting conditions. Imbalance in this circuitry leads to either hyperglycaemia, the hallmark of diabetes mellitus, or hypoglycaemia. Loss of beta cell function coupled to insulin resistance of target tissues, which usually associates with obesity and chronic low-grade inflammation, defines type 2 diabetes [1]; high-grade T lymphocyte inflammation mediating autoimmune beta cell destruction is characteristic of type 1 diabetes [2].

Emerging evidence suggests that alterations in endoplasmic reticulum (ER) function contribute to beta cell disarray in both type 1 and 2 diabetes [3]. In an attempt to restore ER function and prevent apoptosis, cells activate the unfolded protein response (UPR) [4]. The crucial role of the UPR in balancing beta cell death and survival is illustrated in the human Wolfram and Wolcott–Rallison syndromes, in which mutations in UPR genes result in unresolved ER stress, beta cell death and early-onset diabetes [5, 6]. Interestingly, MODY genes such as *Pdx1* and *Hnf1a* regulate UPR-associated genes [7, 8]. These clinical conditions suggest that islet-enriched transcription factors involved in insulin biosynthesis and secretion also preserve the BCM by limiting ER stress.

Paired box (*Pax*) genes encode transcription factors critical for tissue development and cellular differentiation [9]. Paired box 4 (PAX4) is necessary for the generation of pancreatic islet cell progenitors and their differentiation towards beta cells [10, 11]. *Pax4* gene mutations have been associated with type 1 and 2 diabetes as well as with ketosis-prone diabetes, suggesting a key role of PAX4 in mature islets [12, 13]. Accordingly, overexpression of PAX4 in adult beta cells was shown to block streptozotocin (STZ)-induced hyperglycaemia in mice whereas the diabetes-linked variant PAX4R129W was less efficient [14]. Despite differences in nitric oxide synthase 2 (NOS2) levels, both PAX4- and PAX4R129W-expressing islets exhibited similar levels of cytokine-induced NO production, indicating that the nuclear factor-κB (NF-κB) signalling pathway was fully activated and that additional anti-apoptotic pathways are involved in islet survival. Consistent with this premise, PAX4 islets expressed higher levels of B cell CLL/lymphoma 2 (BCL-2) [14]. Nonetheless, overexpression of BCL-2 in islets did not prevent autoimmune-mediated beta cell destruction and development of hyperglycaemia [15]. Thus, although these data highlight the protective function of PAX4 against a chemical acute stress, whether such an effect can also be conveyed in the context of a pathophysiological autoimmune attack and the molecular mechanism involved in this protection remain to be established.

Herein, we investigated whether PAX4 and PAX4R129W could promote beta cell health, preventing the development of hyperglycaemia in the RIP-B7.1 mouse model of experimental autoimmune diabetes (EAD), and sought to characterise the PAX4-regulated pathways implicated in islet survival and expansion.

## Methods

### Animals and bioluminescence imaging

**Fig. 1:**
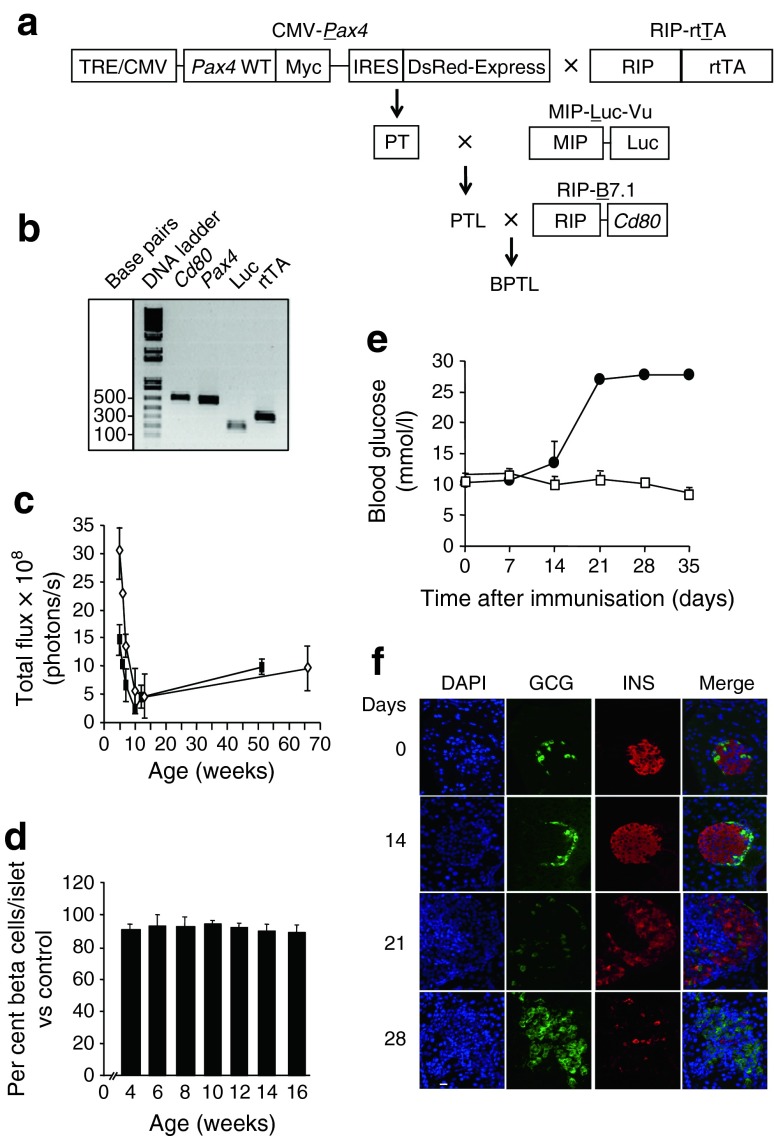
Characterisation of the BPTL mice. (**a**) Schematic diagram showing the generation of the BPTL mouse. (**b**) Genotyping of BPTL mice by PCR using primer sets for *Cd80*, *Pax4*, *Luc* and *rtTA*. (**c**) The bioluminescent signal was correlated with age in BPTL male (white diamonds, *n* = 5) and female (black rectangles, *n* = 5) mice. (**d**) BCM calculated as a percentage of total islet cells and compared with 8-week-old animals was assessed in both sexes. On average, cells from at least 25 islets were counted per animal, with each group comprising at least 5–6 mice. (**e**) Blood glucose levels were measured for up to 35 days in BPTL mice (*n* = 3) immunised (black circles) or not (control, white squares). (**f**) Pancreatic sections of BPTL mice at the indicated time after immunisation were co-immunostained for glucagon (green) and insulin (red). Nuclei were stained with DAPI (blue). Scale bar, 25 μm. GCG, glucagon; INS, insulin

Mouse experiments were approved by the local ethics committee and performed according to the Spanish law on animal use RD 53/2013. The rationale for using RIP-B7.1 (kindly supplied by B. O. Boehm) rather than NOD mice for the current study is provided in the electronic supplementary material (ESM) Methods. BPTL mice were derived as outlined in Fig. 1a, b and maintained on a C57bl/6 background. This mouse harbours four transgenes: (1) RIP-B7.1, a construct coding for the *Cd80* gene under control of the rat insulin promoter (RIP); (2) the tetracycline response element (TRE)/cytomegalo mosaic virus (CMV) *Pax4*, a tetracycline-inducible CMV promoter driving *Pax4* expression; (3) the RIP-reverse tetracycline trans-activator (*rtTA*), a construct allowing selective expression of *Pax4* in beta cells exposed to DOX; and (4) the MIP-Luciferase (*Luc*), a construct that expresses luciferase under the mouse insulin promoter (MIP), allowing assessment of BCM using non-invasive in vivo imaging technology. Induction of *Pax4* or *Pax4*R129W gene expression using doxycycline (DOX) was performed as previously described [14], while EAD was achieved by i.m. immunisation of 9-week-old BPTL animals with 50 μg of pC1/ppins plasmid DNA (1 μg/μl) encoding the murine preproinsulin II. Blood glucose levels were measured using a Precision Xceed glucometer (Abbott, Madrid, Spain). Bioluminescence imaging was performed using a Xenogen IVIS 50 imaging system as previously described [16].

### Islet isolation and treatment

Islets were isolated and cultured as previously described [14]. Total RNA was extracted using the RNeasy Micro Kit (Qiagen, Madrid, Spain) and quantitative (q)-PCR was performed as described previously [17]. Primer sequences can be obtained on request. For studies on thapsigargin-induced ER stress and apoptosis, islets from either *Pax4*/*rtTA* or *Pax4*R129W/*rtTA* transgenic mice were treated with 1 μg/l DOX for 96 h or left untreated [14]. Fluorescence of the *Discosoma* sp. red fluorescent protein (DsRed) correlating with *Pax4* or *Pax4R129W* expression was monitored using an ImageXpress Micro System (Molecular Devices, Wokingham, UK). Islets were then treated or not with 1 μmol/l thapsigargin for 48 h and apoptosis was assessed by ELISA (Roche Diagnostics, Mannheim, Germany). Glucose-stimulated insulin secretion (GSIS) was performed as previously described [18].

### Intracellular Ca^2+^ measurements

Isolated islets were incubated for 1 h at room temperature with 2 μmol/l Fura-2 (Qiagen). Fluorescence recordings were performed using an inverted epifluorescence microscope (Axiovert 200; Zeiss, Jena, Germany) equipped with 360 nm and 380 nm band-pass filters. Recordings were expressed as the ratio of fluorescence at 360 nm and 380 nm (F360/380). Images were taken every 3 s. Intracellular [Ca^2+^] changes in response to stimuli were analysed as previously described [19].

### MIN6 cell culture and RNA interference

MIN6 cells were cultured as previously described [20] and transfected with either 50 μmol *Pax4* small interfering (si)RNA (Sigma) or scramble siRNA using Oligofectamine (Life Technologies, Madrid, Spain). Cells were either processed for RNA or treated with thapsigargin (Sigma-Aldrich, Madrid, Spain) to assess apoptosis 48 h after transfection.

### Immunohistochemistry and electron microscopy

Dissected pancreases were fixed in 4% paraformaldehyde and processed at the Histology Core Facility, Andalusian Center for Molecular Biology and Regenerative Medicine (CABIMER). A detailed immunocytochemistry protocol along with the list of antibodies used is provided in ESM Methods and ESM Table 1. Validation of antibodies was performed on appropriate control samples. The BCM and islet cell number were assessed as described elsewhere [21]. Apoptosis, proliferation and DNA damage were quantified by counting caspase-3-, Ki67- and p53BP1-positive cells, respectively, in at least 20 to 50 islets from three independent pancreatic sections from 3–4 mice per group. Insulitis scoring was performed as previously outlined [22]. For electron microscopy (EM), pancreatic islets were processed using a standard Spurr protocol [23]. Images were acquired with an electron-multiplying charge-coupled device (EMCCD) camera (TRS 2 k × 2 k).

### RNA microarray

Labelled cRNA samples were prepared from pools of at least 100 islets isolated from either *Pax4*/*rtTA* or *Pax4*R121W/*rtTA* transgenic animals (8-week-old females) treated with DOX or not treated [14]. Three preparations of cRNA per group were then hybridised to the GeneChip Mouse Gene 1.0 ST Array chip (Affymetrix, Santa Clara, CA, USA) using the standard protocols of the Genomic Core Facility, CABIMER. Raw data are accessible in the Gene Expression Omnibus database under accession number GSE62846, while its analysis is described in ESM Methods.

### Statistical analysis

Results are expressed as mean ± SEM. Statistical differences between two conditions were estimated using the unpaired Student’s *t* test. One-way ANOVA was used for comparison of more than two groups with Bonferroni post hoc test or non-parametric Mann–Whitney test (*p < 0.05 and **p < 0.01).

## Results

### PAX4 expression blunts EAD in immunised BPTL mice

We first monitored in vivo the bioluminescence intensity (BLI) emitted by beta cells of BPTL mice from 4 to 65 weeks of age (Fig. 1c). In 4-week-old mice, the bioluminescence signal was two fold higher in male mice than females. Consistent with a transient wave of beta cell apoptosis and decreased rate of islet growth around weaning [24], both sexes displayed a decline in BLI signal between the fourth and the ninth week (Fig. 1c). Thereafter, this signal did not significantly change with age (Fig. 1c). Morphometric evaluation revealed that the volume density of beta cells was similar in animals aged from 4 to 16 weeks (Fig. 1d). Thus, by 9 weeks of age the bioluminescent signal reflects the mass and function of beta cells, which we hereafter refer to as the functional beta cell mass (BCM). Similar to the RIP-B7.1 animal, immunised BPTL mice developed hyperglycaemia within 21 days (Fig. 1e) due to a gradual loss of beta cells (Fig. 1f), whereas non-immunised mice remained normoglycaemic (Fig. 1e, f).

**Fig. 2:**
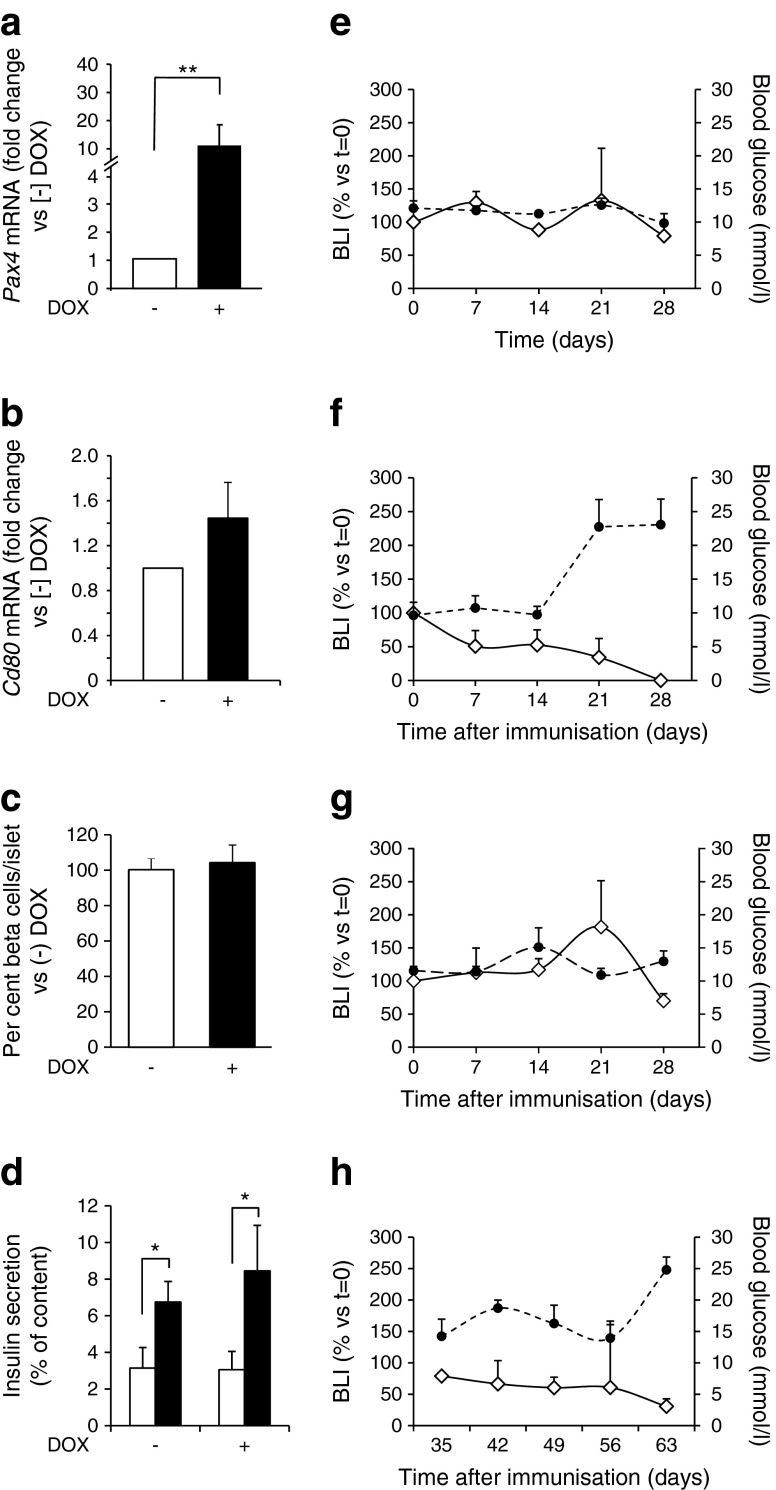
PAX4 prevents the development of hyperglycaemia in immunised BPTL mice. Islet (**a**) *Pax4* and (**b**) *Cd80* transcript levels in 5-week-old BPTL animals treated with DOX for 1 month or not treated (*n* = 4–6). Relative mRNA levels were normalised to the transcript levels of the housekeeping gene β-actin and/or *Rps29*. Data were calculated as fold change compared with non-DOX-treated mice. **p < 0.01. (**c**) BCM was calculated as a percentage of total islet cells and compared with the (−) DOX group. On average, cells from at least 25 islets were counted per animal (*n* = 4–6). (**d**) Insulin secretion by isolated BPTL islets treated or not with DOX was assessed in 30 min static incubations in response to glucose at 2.5 mmol/l (white bars) and 16.5 mmol/l (black bars). Insulin released was quantified by ELISA and expressed as a percentage of total cellular insulin content. *n =* 4–7, *p < 0.05. BLI (white diamonds) and blood glucose levels (black circles) was measured weekly for up to 4 weeks in (**e**) control untreated BPTL mice (*n* = 4), (**f**) immunised non-DOX-treated (*n* = 6) and (**g**) immunised DOX-treated BPTL animals (*n* = 6). (**h**) BLI and blood glucose level measurements were extended to 63 days in immunised and DOX-treated BPTL mice (*n* = 6). BLI results are presented as per cent change compared with day 0 (*t* = 0), while blood glucose levels are expressed as means ± SEM

**Fig. 3:**
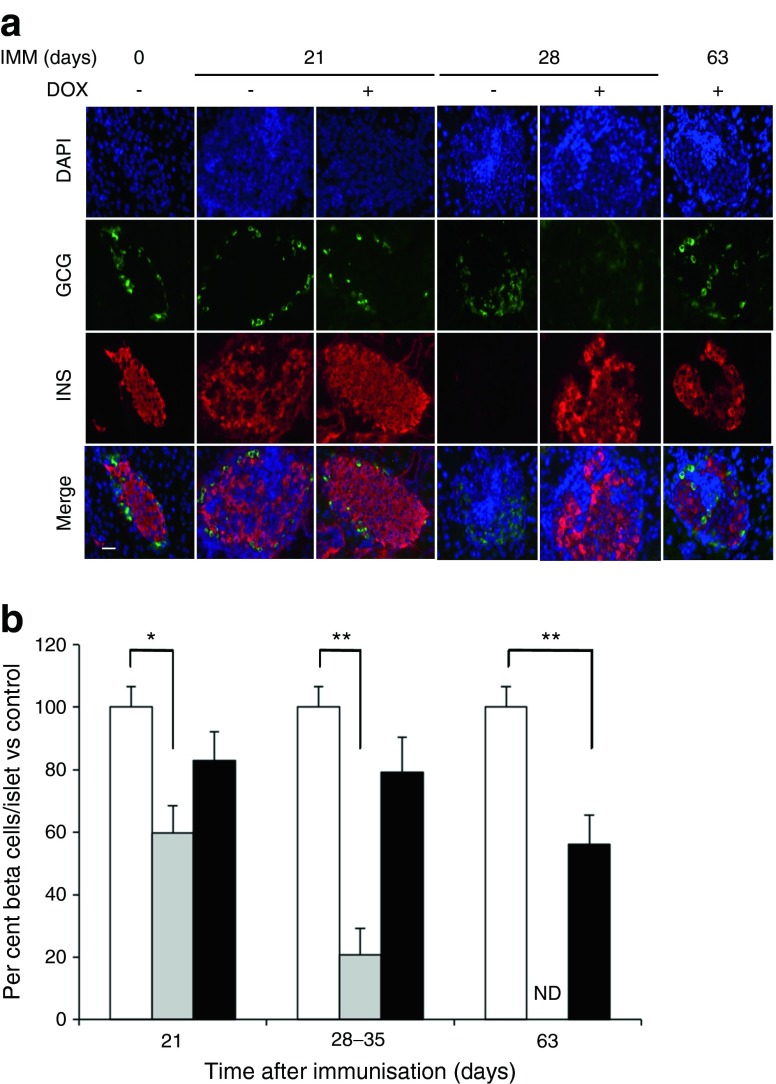
PAX4 preserves the functional BCM in immunised BPTL mice. (**a**) Immunohistochemical analysis of glucagon (green) and insulin (red) in pancreases from BPTL mice treated or not with DOX and killed at days 0, 21, 28 and 63 post-immunisation. Nuclei are stained blue. Scale bar, 25 μm. (**b**) BCM in control non-immunised and non-DOX-treated (white bars), immunised and non-DOX-treated (grey bars) and immunised and DOX-treated (black bars) mice was calculated as a percentage of total islet cells and compared with the control group at each time point. On average, cells from at least 25 islets were counted per animal (*n* = 5–6). As similar results were obtained for days 28 and 35, the data were combined as a single time point. ND, not determined, as this group was killed at day 35 to comply with animal welfare guidelines. *p < 0.05 and **p < 0.01. GCG, glucagon; IMM, immunised; INS, insulin

We next determined whether PAX4 expression could prevent EAD. Five-week-old BPTL mice were treated with DOX for 4 weeks prior to immunisation. Compared with control mice, islets from treated mice revealed a ten fold increase in *Pax4* expression (Fig. 2a), but no change in *Cd80* transcript levels (Fig. 2b). DOX treatment did not alter the BCM or GSIS of PAX4-expressing islets (Fig. 2c, d). Nine-week-old untreated controls remained normoglycaemic and featured no variation in bioluminescent signal for up to 28 days (Fig. 2e). Immunised BPTL mice without DOX revealed a rapid decrease in BLI, reaching undetectable levels by day 28 post-immunisation, which coincided with sustained hyperglycaemia (Fig. 2f). Escalation in blood glucose level observed 21 days after immunisation correlated with a 60% decrease in BLI. DOX-treated and immunised BPTL mice maintained both normoglycaemia and bioluminescent signal (Fig. 2g). Protection was extended up to 63 days (Fig. 2h), at which point 65% of immunised and DOX-treated BPTL mice developed hyperglycaemia, probably because of the robust immune attack conveyed by CD80 overexpression. Immunised and DOX-treated RIP-B7.1 mice developed hyperglycaemia (ESM Fig. 1), excluding a protective effect mediated by the antibiotic through alteration in the gut microbiome [25, 26]. Non-DOX-treated BPTL mice suffered a 40% and 80% reduction in the functional BCM after 21 and 28 days of immunisation, respectively. Such changes were not observed in DOX-treated mice (Fig. 3a, b). By day 63, the latter animals retained approximately 50% of the functional BCM (Fig. 3b).

### PAX4 improves beta cell health and mitigates the autoimmune attack

**Fig. 4:**
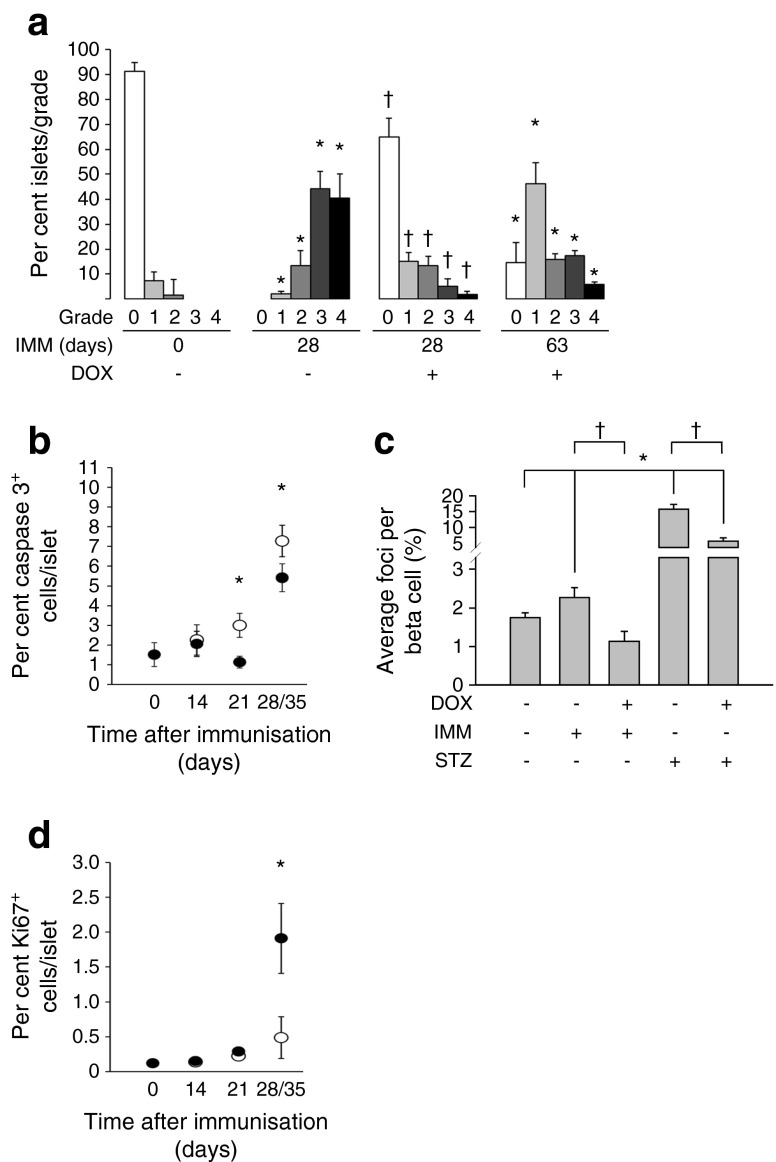
BPTL mice overexpressing PAX4 display reduced insulitis, correlating with decreased islet apoptosis and DNA damage and increased proliferation. (**a**) Insulitis was scored as grade 0–4 according to the percentage of infiltrated islet area (0: 0%; 1: <10%; 2: >10% and <55%; 3: >55% and <75%; 4: >75%). *n* = 5–6, *p < 0.05 vs immunised day 0 and (−) DOX group; ^†^ *p* < 0.05 vs immunised day 28 and (−) DOX group. (**b**) Apoptosis was assessed by immunohistochemical analysis of cleaved caspase-3 in pancreatic islets from BPTL mice treated (black ovals) or not (white ovals) with DOX and killed at the time points. As similar results were obtained for days 28 and 35, the data were combined as a single time point. *n* = 5–6, *p < 0.05, (+) vs (−) DOX groups within each time point. (**c**) The average number of 53BP1 foci per beta cell was assessed from pancreatic islets of either 14-day-old immunised BPTL mice or 2-day-old STZ-treated PAX4 mice exposed (+) or not (−) to DOX. *n* = 5–6, *p < 0.05 vs untreated mice; ^†^ *p* < 0.05 vs DOX-untreated immunised or STZ-treated mice. (**d**) Cell proliferation was assessed by the combined immunohistochemical analysis of Ki67 in pancreatic islets from BPTL mice treated (black ovals) or not (white ovals) with DOX and killed at the time points. *n* = 5–6, *p < 0.05, (+) vs (−) DOX groups within the time point. IMM, immunised

Insulitis assessment at 28 days after immunisation revealed that 65% of islets of DOX-treated BPTL mice were insulitis free (grade 0), whereas 90% of islets derived from non-DOX-treated BPTL mice displayed severe insulitis (grades 2–4) (Fig. 4a). Even at 63 days after immunisation only 50% of islets from DOX-treated BPLT mice displayed mild insulitis (Fig. 4a). DOX treatment also diminished the percentage of cleaved CASPASE-3-positive islet cells in immunised animals (Fig. 4b and ESM Fig. 2a). DNA damage induced by NO and reactive oxygen species (ROS) contributes to beta cell death [27, 28]. Immunostaining for the double-strand DNA break marker p53BP1 revealed that PAX4 overexpression reduced DNA damage in islets from either immunised BPTL or STZ-treated mice (Fig. 4c). These changes were paralleled by increased cell proliferation in DOX-treated BPLT islets (Fig. 4d and ESM Fig. 2b).

### PAX4 regulates genes important for beta cell proliferation and ER homeostasis

**Fig. 5:**
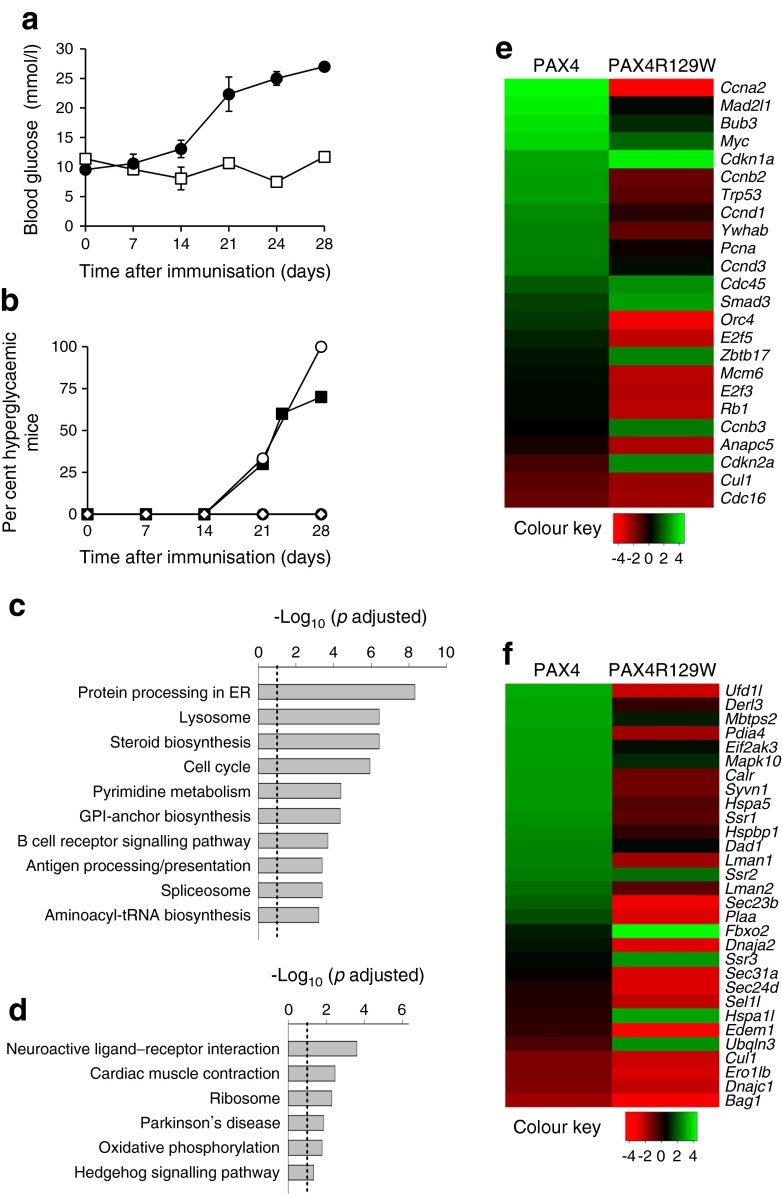
ER homeostasis and cell cycle are key cellular pathways targeted by PAX4. (**a**) Blood glucose levels were measured for up to 28 days in control non-DOX-treated (control, white squares) and immunised DOX-treated (black circles) mutBPTL mice (*n* = 4). (**b**) Hyperglycaemia incidence was assessed in control non-treated (*n* = 5, white diamonds), immunised and non-DOX-treated (*n* = 6, white circles) and immunised and DOX-treated (*n* = 6, black circles) BPTL mice as well as in immunised and DOX-treated mutBPTL mice (*n* = 7, black squares). (**c**, **d**) Significantly enriched KEGG pathways (adjusted *p* value <0.05) altered by (**c**) PAX4 and (**d**) PAX4R129W. The dotted line shows the threshold of significance. (**e**, **f**) Heat maps displaying *t* statistic values of (**e**) cell cycle and (**f**) protein processing in the ER KEGG pathway genes modulated in either PAX4 or PAX4R129W islets vs control. Colours display the *t* statistic values for all genes within the corresponding KEGG pathway, estimated using the *t* statistic value from differential expression analysis. GPI, glycosylphosphatidylinositol

Transcriptome profiling was conducted on PAX4 and PAX4R129W islets to highlight PAX4 target genes involved in beta cell health, and those altered by the diabetes-linked mutant variant R129W. We initially demonstrated that DOX-treated and immunised mutBPTL mice developed hyperglycaemia (Fig. 5a) with an incidence of approximately 75% by day 28 compared with 100% in non-DOX-treated immunised animals (Fig. 5b). Transcriptome analysis revealed that 770 transcripts were upregulated and 449 were downregulated in PAX4 islets, whereas 1437 genes were upregulated and 1136 downregulated in PAX4R129W islets. Genes showing the largest changes are listed in ESM Table 2. Notwithstanding this list, we substantiated by qPCR our previous findings [14] that *Mafa* transcript levels were repressed whereas *Nos2* levels were unchanged in PAX4 islets (ESM Fig. 3). However, *Bcl2* levels were not increased in PAX4-overexpressing islets (ESM Fig. 3), arguing against a role of this factor in protecting from EAD [15].

A functional enrichment analysis disclosed that the cell cycle and the protein processing in ER pathways were among the top upregulated Kyoto Encyclopaedia of Genes and Genomes (KEGG) pathways in islets overexpressing PAX4, but were among the most significantly downregulated pathways in islets overexpressing PAX4R129W (adjusted *p* values < 0.05) (Fig. 5c, d and ESM Tables 3 and 4). To contrast the expression levels of genes contributing to these pathways, we generated heat maps amenable to statistical analysis (raw *p* value < 0.05). Several genes associated with cell cycle were increased in PAX4 islets, while remaining unchanged or having lower levels in islets expressing PAX4R129W (Fig. 5e). Genes encoding proteins for peptide folding (*Hspa5* [also known as *Bip*], *Calr*), ER–Golgi translocation (*Lman1*, *Lman2*, *Sec23b* and *Plaa*) and ER-associated protein degradation (ERAD) (*Ufd1l*, *Derl3*, *Pdia4*, *Ssr3*, *Syvn1* and *Dnaja2*/*Hsp40*) were upregulated after overexpression of PAX4, but were downregulated after overexpression of PAX4R129W (Fig. 5f). The UPR-associated genes *Mbtps2*, *Eif2ak3* and *Mapk10* were upregulated in PAX4 islets and were marginally altered in PAX4R129W islets (Fig. 5f).

### PAX4 targets ER integrity and calcium homeostasis, protecting cells against apoptosis

**Fig. 6:**
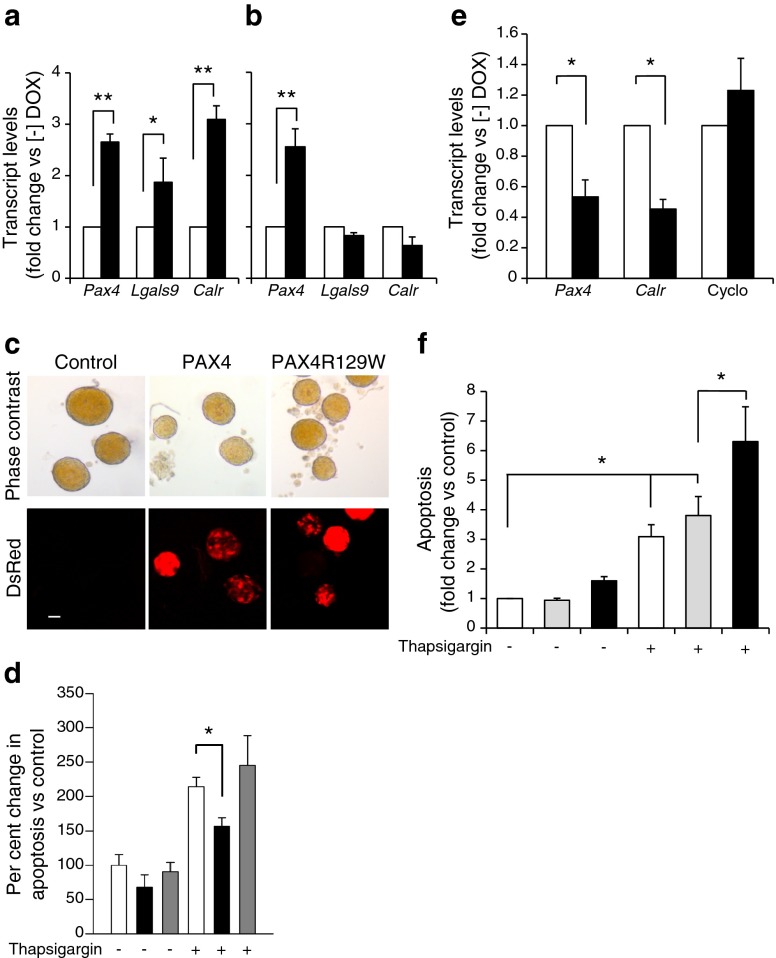
PAX4-regulated ER homeostasis prevents cell degeneration. Islet PAX4, galectin-9 (*Lgals9*) and calreticulin (*Calr*) transcript levels were assessed in (**a**) PAX4 or (**b**) PAX4R129W transgenic mice treated (black bars) or not treated (white bars) with DOX for 1 month. The relative mRNA levels were normalised to transcript levels of the housekeeping gene β-actin. Data were calculated as fold change compared with the values in control, non-DOX-treated animals. *n* = 3, **p < 0.01 and *p < 0.05. (**c**) *Pax4* or *Pax4*R129W induction in DOX-treated islets was qualitatively assessed through DsRed fluorescence. Scale bar, 10 μm. (**d**) Control (white bars), PAX4-expressing (black bars) and PAX4R129W-expressing (grey bars) islets were then challenged with 1 μmol/l thapsigargin or not challenged and apoptosis was measured by ELISA. Results expressed as per cent change compared with control non-thapsigargin-treated islets. *n* = 5, *p < 0.05. (**e**) *Pax4*, *Calr* and cyclophylin expression levels were measured in MIN6 cells treated with siControl (white bars) and si*Pax4* (black bars). mRNA levels were normalised to the transcript levels of the housekeeping gene β-actin. Data were calculated as fold change compared with the values in siControl-treated cells. *n* = 4, *p < 0.05. (**f**) Apoptosis was evaluated in control MIN6 (white bars), and cells treated with siControl (grey bars) or si*Pax4* (black bars), untreated or treated with thapsigargin. Results are expressed as fold change compared with control non-thapsigargin-treated cells. *n* = 5, *p < 0.05

Given that PAX4 but not PAX4R129W targets genes that are involved in ER homeostasis, we investigated the contribution of ER to PAX4-mediated beta cell health. We initially assessed expression levels of calreticulin (*Calr*), a major Ca^2+^ binding protein of the ER lumen and of galectin-9 (*Lgals9*), a protein involved in immune modulation. Although *Pax4* and *Pax4*R129W transcript levels were increased three fold, *Calr* and *Lgals9* expression levels were only increased in PAX4 islets (Fig. 6a, b). As Ca^2+^ handling by the ER affects the cell sensitivity to apoptosis, we assessed whether PAX4 and PAX4R129W could protect against ER-stress-induced beta cell apoptosis. Islets treated in vitro with DOX exhibited DsRed fluorescence, confirming the expression of the transgenes (Fig. 6c). Thapsigargin exposure prompted a two fold increase in apoptosis in control mice (Fig. 6d). DOX reduced both basal and thapsigargin-induced apoptosis only in PAX4 islets (Fig. 6d). However, *Pax4* siRNA-mediated repression in MIN6 cells caused a 60% decrease in *Calr* transcript levels, with a concomitant sensitisation to thapsigargin-induced apoptosis compared with siControl and thapsigargin-treated cells (Fig. 6e, f).

**Fig. 7:**
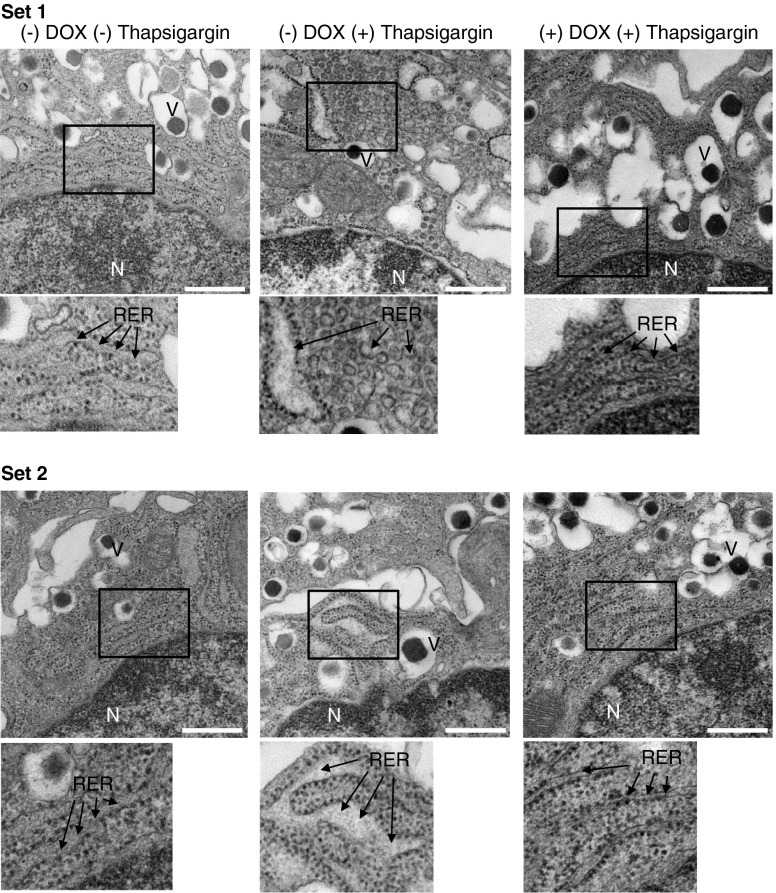
ER integrity is preserved in PAX4-overexpressing islets. Two sets (upper and lower) of beta cell micrographs from islets isolated from PAX4 transgenic mice, treated with DOX or not treated, and challenged with 1 μmol/l thapsigargin or not challenged. Arrows indicate the RER. Inset is enlarged below each image. Scale bar, 0.5 μm. N, nucleus; V, insulin vesicles

**Fig. 8:**
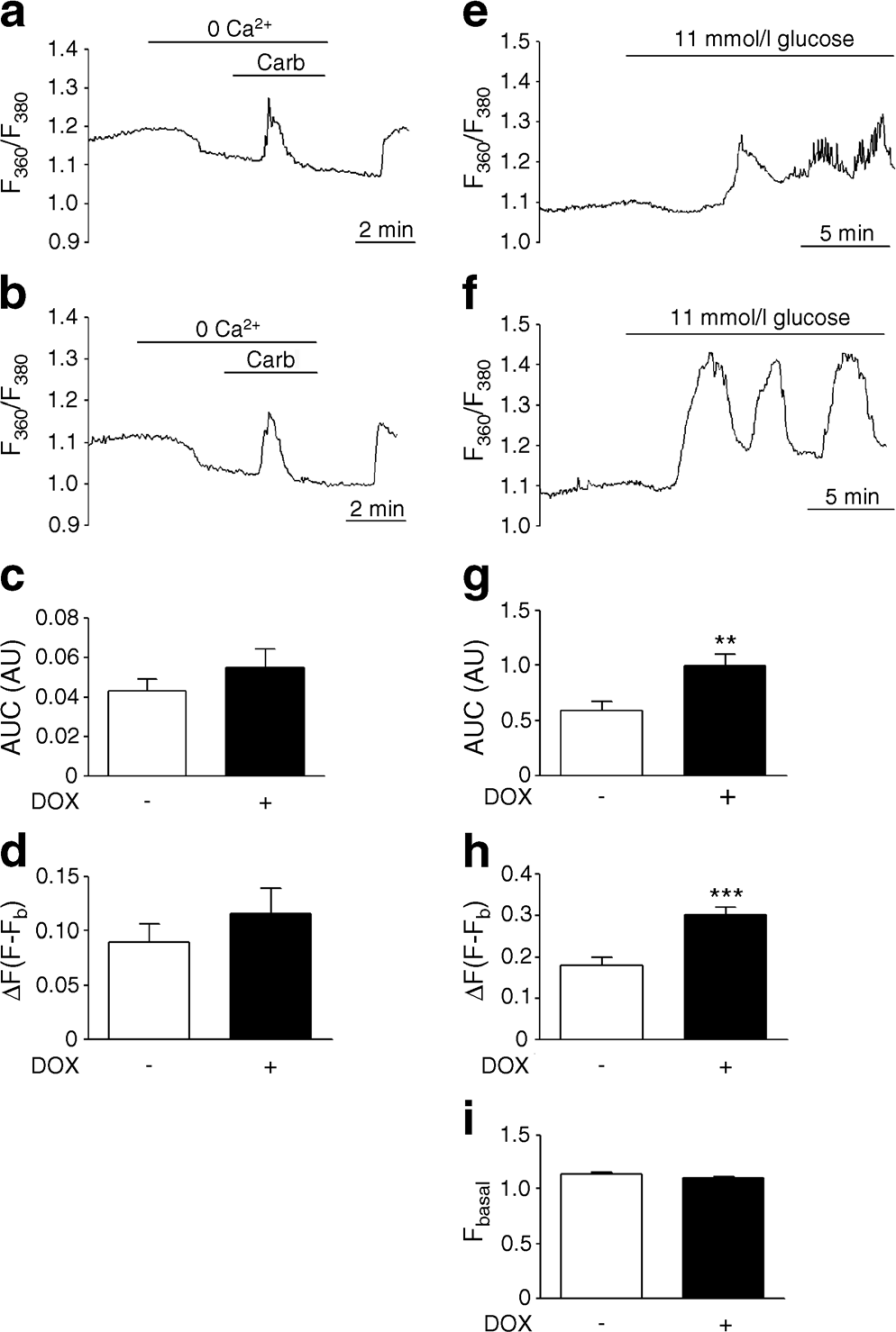
PAX4-overexpressing islets exhibit normal calcium oscillation in response to glucose. Carbachol-stimulated (100 μmol/l) intracellular release of Ca^2+^ was measured in intact islets (**a**) treated with DOX or (**b**) not treated. As indicated by the bars, islets were exposed to Ca^2+^-deficient medium containing 2 mmol/l EGTA prior to applying 100 μmol/l carbachol. (**c**) Analysis of the AUC for the time of the stimulus with carbachol from experiments shown in (**a**) and (**b**). (**d**) Analysis of the Ca^2+^ peak amplitude in response to carbachol challenge. (*n* = 5–6). Representative Ca^2+^ signals in response to 11 mmol/l glucose measured in intact islets (**e**) treated with DOX or (**f**) not treated. (**g**) Analysis of the AUC for the last 10 min of the glucose stimulus recording shown in (**e**) and (**f**). (**h**) Analysis of the amplitude of the first Ca^2+^ transient in response to the glucose challenge. (**i**) Analysis of the basal fluorescence during the first minute of the experiments. *n* = 9; **p ≤ 0.01 and ***p ≤ 0.001. ΔF; fluorescence increase

Perturbation in beta cell ER homeostasis has been linked to ER dilation [29, 30]. Accordingly, islets from non-DOX-treated *Pax4* transgenic mice exposed in vitro to thapsigargin displayed distension and fragmentation of the rough ER (RER) in beta cells, an effect prevented by PAX4 overexpression (Fig. 7). ER calcium release in response to carbachol was then measured. Calcium release in PAX4-overexpressing beta cells from isolated islets was marginally higher than that of controls cells (Fig. 8a–d). As the higher ER-Ca^2+^-buffering capacity induced by PAX4 could mask the carbachol-stimulated calcium release, we measured cytosolic Ca^2+^ signals in response to glucose. Glucose-induced calcium oscillations were improved in isolated islets overexpressing PAX4 compared with control islets (Fig. 8e, f). The overall Ca^2+^ signal was also higher in PAX4-overexpressing cells (Fig. 8g–i). Thus, PAX4 overexpression leads to improve ER and Ca^2+^ homeostasis in the face of beta cell stress as encountered during the islet isolation procedure or an immune attack.

## Discussion

Inflammation is a common denominator in types 1 and 2 diabetes, and leads to beta cell failure and death, predominantly by apoptosis. As yet, there is no ‘unifying hypothesis’ for the mechanisms triggering beta cell deterioration [31, 32]; deciphering the molecular roadmap regulated by factors such as PAX4—mutations in which are linked to both forms of diabetes—may help identify common pathways. Herein, we provide proof-of-concept that PAX4, but not PAX4R129W, preserves the BCM and delays the development of hyperglycaemia in the RIP-B7.1 mouse model of EAD. This highlights the mechanistic differences between the wild type and mutant variant.

Consistent with previous reports exploiting the RIP-B7.1 model, 90–100% of immunised non-DOX-treated RIP-B7.1 mice bearing either the *Pax4* or *Pax4R129W* transgene developed hyperglycaemia within 3 weeks, correlating with insulitis and beta cell destruction [33]. DOX-mediated induction of *Pax4* prevented hyperglycaemia development in BPTL animals up to 4 weeks after immunisation, whereas *Pax4R129W* induction partially reduced the hyperglycaemic incidence in mutBPTL mice. Thus, PAX4 blunts hyperglycaemia in an autoimmune context, in contrast to several other factors such as caspase-3-generated RAS p21 protein activator 1 (RasGAP) N-terminal fragment (fragment N), cytokine response modifier A (CRMA) or BCL-2 which, despite increasing BCM, could not prevent hyperglycaemia in animal models of type 1 diabetes [15, 22, 34]. Our data imply that in addition to inhibiting apoptosis, PAX4 is involved in additional regulatory pathways, possibly including immune modulation. Correspondingly, insulitis was reduced after PAX4 overexpression, an effect not attributable to a non-specific repression of the *Cd80* transgene that facilitates the immune response. Our observations extend the analogous findings that inhibition of vascular endothelial growth factor receptor 2 (VEGFR-2) in NOD mice reversed hyperglycaemia by abrogating insulitis and restoring islet cell function [35]. Although the mechanism by which PAX4 acts at the interface of beta cells and the immune system to blunt insulitis and improve islet recovery remains undefined, our genetic analysis revealed that *Lgals9* was specifically upregulated in PAX4 islets. This gene induces apoptosis of differentiated T helper 1 (Th1) cells [36], and its overexpression in NOD mice reduced insulitis and hyperglycaemia [37], prolonging the survival of grafts [38]. It is therefore plausible that, by enhancing *Lgals9* expression, PAX4 may downregulate Th1 function, partially impeding insulitis and improving islet survival.

Transcriptome profiling revealed that cell-cycle-associated genes upregulated by PAX4 were downregulated by PAX4R129W, despite comparable expression levels of both transgenes. Specifically, the type 2 diabetes-associated cyclin-dependent kinase inhibitor 2A, which strongly inhibits the proliferative cyclin-dependent kinase 4 (CDK4) [39], was enriched in PAX4R129W islets whereas it was decreased in PAX4 islets. Reciprocally, cyclin D3, which promotes beta cell survival, was increased in PAX4- but not in PAX4R129W-overexpressing islets [40]. These findings provide some molecular insights into the pathogenic effect of the R129W mutation on beta cell plasticity.

Our data also reveal that PAX4, but not PAX4R129W, is a key regulator of ER function by a combined targeting of genes involved in UPR, Ca^2+^ homeostasis, ER–Golgi translocation, and ERAD. The functional importance of the transcriptional changes in these genes was validated by the capacity of PAX4 to prevent thapsigargin-induced ER ultrastructural abnormalities and apoptosis of beta cells, consistent with the finding that PAX4-binding sites are enriched within the promoter region of palmitate-modified ER stress response genes [41]. In contrast, repression of PAX4-sensitised MIN6 cells to thapsigargin cell death correlates with reduced CALR levels. Calreticulin is a Ca^2+^ chaperone of the ER, which contributes to the quality control of protein folding [42]. Overexpression of CALR in MIN6 cells enhances ER Ca^2+^ stores and prevents NO-induced apoptosis [43]. In this context, the production of NO and ROS induced by inflammatory cytokines in the diabetic environment promotes beta cell death by a variety of mechanisms, including the induction of irreversible double-strand DNA breaks [27, 44]. We found that PAX4 blunts DNA damage in two models of experimental diabetes, pointing to a general protective mechanism, possibly through preserved ER homeostasis, implicating CALR [43]. The finding that PAX4-overexpressing islets exhibited improved glucose-induced Ca^2+^ oscillations points to this premise. Loss of oscillatory capacity is associated with diminished islet glucose sensitivity and increased ER dysfunction [45]. Thus, amplified Ca^2+^ content in the CALR-enriched ER of PAX4-overexpressing beta cells may impact cytosolic calcium dynamics [43, 46], thereby preventing activation of downstream apoptotic pathways under stress conditions.

We conclude that PAX4 favours beta cell survival and regeneration in various deleterious inflammatory and high-grade inflammatory environments, such as autoimmunity, through the coordinated regulation of immune modulation, cell cycle, cell survival, ER homeostasis and DNA repair. While both PAX4 and PAX4R129W modulate these pathways, it is the wild-type transcription factor that conveys pro-survival properties by increasing the expression of selected adaptive genes.

## Electronic supplementary material

ESM Methods (PDF 63 kb)

ESM Fig. 1 (PDF 48 kb)

ESM Fig. 2 (PDF 440 kb)

ESM Fig. 3 (PDF 61 kb)

ESM Table 1 (PDF 50 kb)

ESM Table 2 (PDF 43 kb)

ESM Table 3 (PDF 62 kb)

ESM Table 4 (PDF 51 kb)

## Abbreviations

BCL-2: B cell CLL/lymphoma 2
BCM: Beta cell mass
BLI: Bioluminescence intensity
BPTL: RIP-B7.1/*P**ax4*/*rt**T**A*/MIP-*L**uc*
CABIMER: Andalusian Center for Molecular Biology and Regenerative Medicine
CALR: Calreticulin
DOX: Doxycycline
DsRed: *Discosoma* sp. red fluorescent protein
EAD: Experimental autoimmune diabetes
EM: Electron microscopy
ER: Endoplasmic reticulum
ERAD: ER-associated protein degradation
GSIS: Glucose-stimulated insulin secretion
KEGG: Kyoto Encyclopaedia of Genes and Genomes
Luc: Luciferase
MIP: Mouse insulin promoter
PAX4: Paired box 4
RER: Rough ER
RIP: Rat insulin II promoter
rtTA: Reverse tetracycline-controlled transactivator
si: Small interfering
STZ: Streptozotocin
Th1: T helper 1
UPR: Unfolded protein response
