# Supplementary material for: PAX4 preserves endoplasmic reticulum integrity preventing beta cell degeneration in a mouse model of type 1 diabetes mellitus
Source: Diabetologia. 2016 Jan 26;59:755–65. doi: 10.1007/s00125-016-3864-0 (PMC4779135; doi:10.1007/s00125-016-3864-0)
Supplement: Supplementary file 1 — (PDF 63 kb) [file 125_2016_3864_MOESM1_ESM.pdf]

ESM Methods

The RIP-B7.1 animal model of experimental autoimmune diabetes (EAD)

The transgenic RIP-B7.1 mouse express the B7 co-stimulatory molecule specifically in beta cell specific. B7 expression overcomes a critical checkpoint in T cell tolerance, thereby inducing EAD after immunisation with a preproinsulin II cDNA encoding plasmid [1, 2]. The RIP-B7.1 model was preferred to the NOD model [3] because of a higher incidence of CD8 T-cell autoimmune-mediated beta cell destruction without gender preponderance and because its insulitis resembles that seen in type 1 diabetes patients [2, 4].

Immunohistochemistry

Pancreas sections of 7 µm thickness were rehydrated by immersion in decreasing concentration of ethanol. Heat induced antigen retrieval was performed in 0.01M Sodium Citrate Buffer (pH6). In the case of Ki67 and cleaved caspase-3 antigen retrieval was carried out using a pressure cooker. Thereafter, all sections were maintained overnight at 4ºC in the presence of primaries antibodies at the indicated dilutions (ESM Table 1). Subsequently, corresponding secondary antibodies (ESM Table 1) were added for 1 h at room temperature, followed by DAPI-nuclear staining (Life Technologies), slides were finally cover slipped using a fluorescent mounting medium (DAKO, Barcelona, Spain).

Bioinformatics analysis

The Robust Multiarray Analysis (RMA) method was applied on a per-chip basis for background correction [5]. Subsequent normalization across arrays and summarization were performed using a quantile algorithm and median-polish, respectively [6] via *oligo* package from Bioconductor (http://www.bioconductor.org). A differential gene expression analysis was then performed using the *limma* package [7]. Computed p-values were corrected using the widely accepted False Discovery Rate (FDR) method to harmonize for the multiple comparisons for all the genes [8]. Gene set analysis was achieved for KEGG pathways using the logistic regression model [9] while KEGG annotations for genes in the microarrays were extracted from the Reactome database [10]. Heatmap displaying t-statistic values of differential expression analyses for Pax4 (+DOX versus -DOX) and Pax4R129W (+DOX versus -DOX) were then generated with selected genes of either Pax4 or Pax4R129W associated with the statistically enriched (p-adjusted<0.05) cell cycle and protein processing in ER KEGG pathways.

References

[1] Karges W, Pechhold K, Al Dahouk S, et al. (2002) Induction of autoimmune diabetes through insulin (but not GAD65) DNA vaccination in nonobese diabetic and in RIP-B7.1 mice. Diabetes 51: 3237-3244

[2] Karges W, Rajasalu T, Spyrantis A, Wieland A, Boehm B, Schirmbeck R (2007) The diabetogenic, insulin-specific CD8 T cell response primed in the experimental autoimmune diabetes model in RIP-B7.1 mice. Eur J Immunol 37: 2097-2103

[3] Anderson MS, Bluestone JA (2005) The NOD mouse: a model of immune dysregulation. Annu Rev Immunol 23: 447-485

[4] Fiorina P (2010) beta-Cells step up in controlling the autoimmune response. Diabetes 59: 1861-1864

[5] Irizarry RA, Hobbs B, Collin F, et al. (2003) Exploration, normalization, and summaries of high density oligonucleotide array probe level data. Biostatistics 4: 249-264

[6] Carvalho BS, Irizarry RA (2010) A framework for oligonucleotide microarray preprocessing. Bioinformatics 26: 2363-2367
[7] Smyth GK (2004) Linear models and empirical bayes methods for assessing differential expression in microarray experiments. Stat Appl Genet Mol Biol 3: Article3
[8] Benjamini Y, Hochberg Y (1995) Controlling the False Discovery Rate: A Practical and Powerful Approach to Multiple Testing. J Roy Statist Soc Ser B 57: 289-300
[9] Montaner D, Dopazo J (2010) Multidimensional gene set analysis of genomic data. PLoS One 5: e10348
[10] Matthews L, Gopinath G, Gillespie M, et al. (2009) Reactome knowledgebase of human biological pathways and processes. Nucleic Acids Res 37: D619-622
