# Supplementary material for: PAX4 preserves endoplasmic reticulum integrity preventing beta cell degeneration in a mouse model of type 1 diabetes mellitus
Source: Diabetologia. 2016 Jan 26;59:755–65. doi: 10.1007/s00125-016-3864-0 (PMC4779135; doi:10.1007/s00125-016-3864-0)
Supplement: Supplementary file 2 — (PDF 48 kb) [file 125_2016_3864_MOESM2_ESM.pdf]

ESM Fig. 1

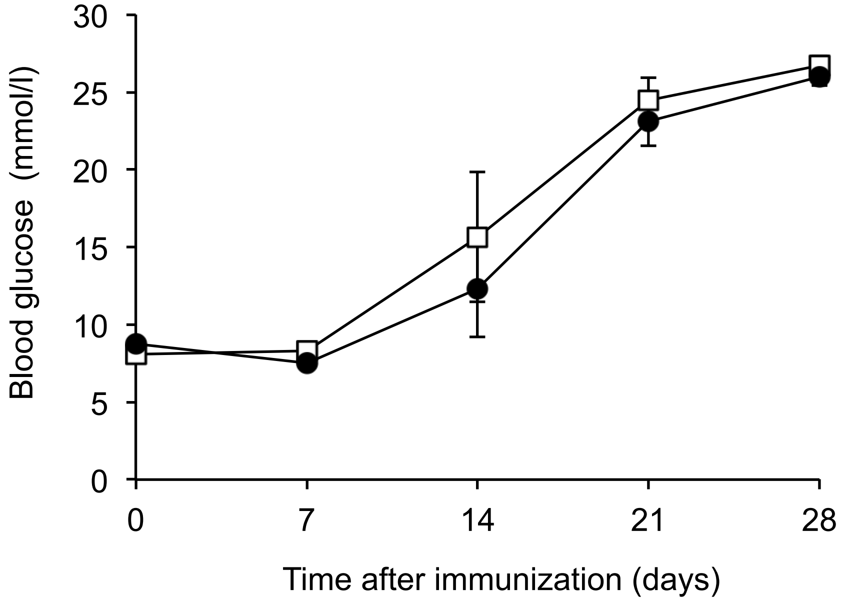

ESM Fig.1. Development of hyperglycaemia in immunised RIP-B7.1 mice is not altered by DOX treatment. Blood glucose levels were measured for up to 28 days in immunised RIP-B7.1 mice continuously treated (black circles) or not (white squares) with DOX. *n=4.*
