# Supplementary material for: PAX4 preserves endoplasmic reticulum integrity preventing beta cell degeneration in a mouse model of type 1 diabetes mellitus
Source: Diabetologia. 2016 Jan 26;59:755–65. doi: 10.1007/s00125-016-3864-0 (PMC4779135; doi:10.1007/s00125-016-3864-0)
Supplement: Supplementary file 3 — (PDF 440 kb) [file 125_2016_3864_MOESM3_ESM.pdf]

# ESM Fig. 2

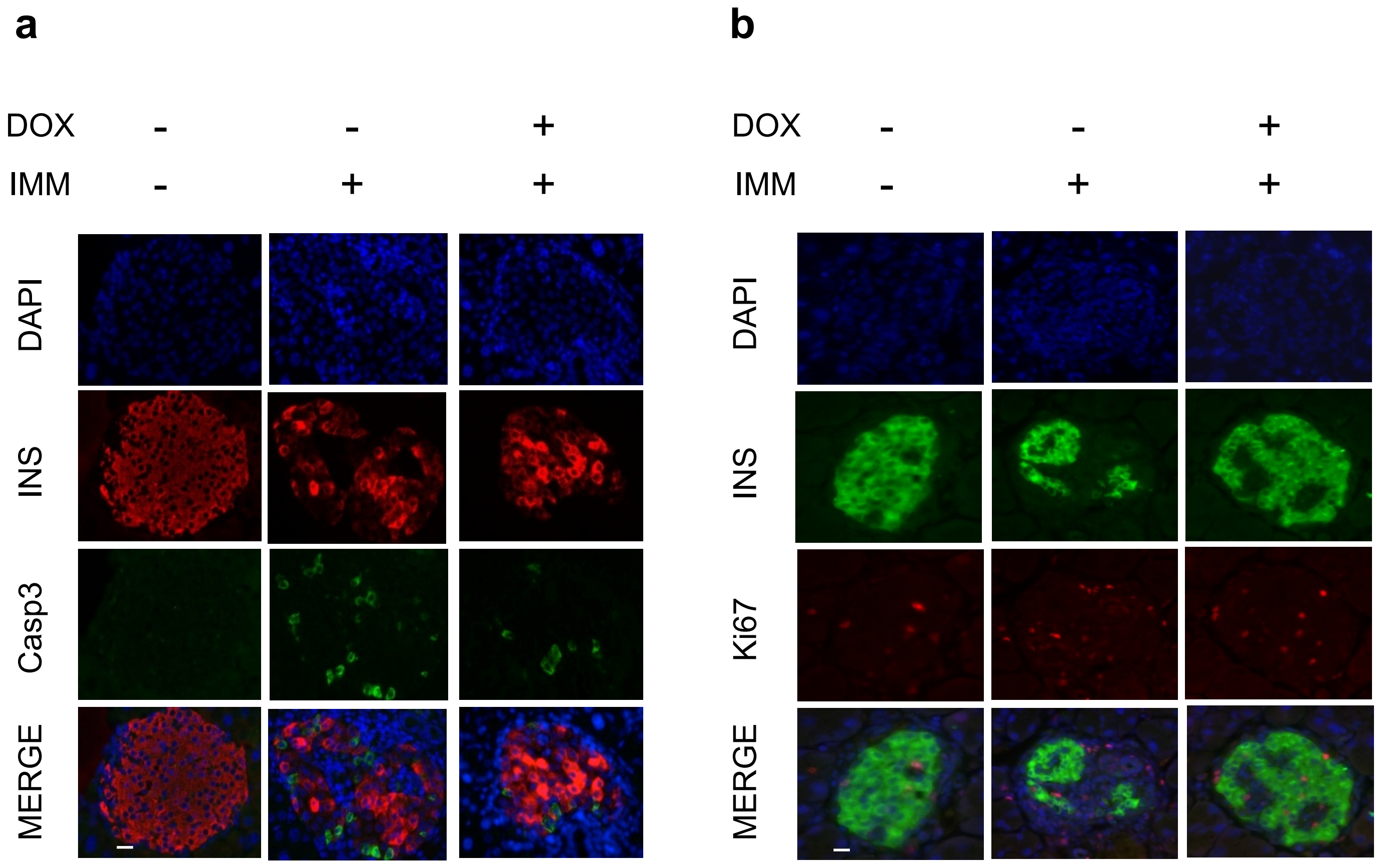


**ESM Fig.2. Assessment of islet cell apoptosis and proliferation in BPTL mice by immunohistochemistry. (a)** Apoptosis detection by immunohistochemical analysis of cleaved CASPASE-3 (Casp3, green) and INSULIN (red) on pancreatic paraffin sections from control, immunized (IMM) or immunized and DOX-treated BPTL mice. Nuclei were stained with DAPI (blue). Representative images correspond to animals killed at 28 to 35 days post immunization. **(b)** Cell proliferation assessment by immunohistochemical analysis of Ki67 (red) and INSULIN (green) on pancreatic paraffin sections from control, immunized (IMM) or immunized and DOX-treated BPTL mice. Nuclei were stained with DAPI (blue). Representative images correspond to animals killed at 28 to 35 days post immunization. Bar, 25 μm.
