# Supplementary material for: PAX4 preserves endoplasmic reticulum integrity preventing beta cell degeneration in a mouse model of type 1 diabetes mellitus
Source: Diabetologia. 2016 Jan 26;59:755–65. doi: 10.1007/s00125-016-3864-0 (PMC4779135; doi:10.1007/s00125-016-3864-0)
Supplement: Supplementary file 4 — (PDF 61 kb) [file 125_2016_3864_MOESM4_ESM.pdf]

ESM Fig. 3

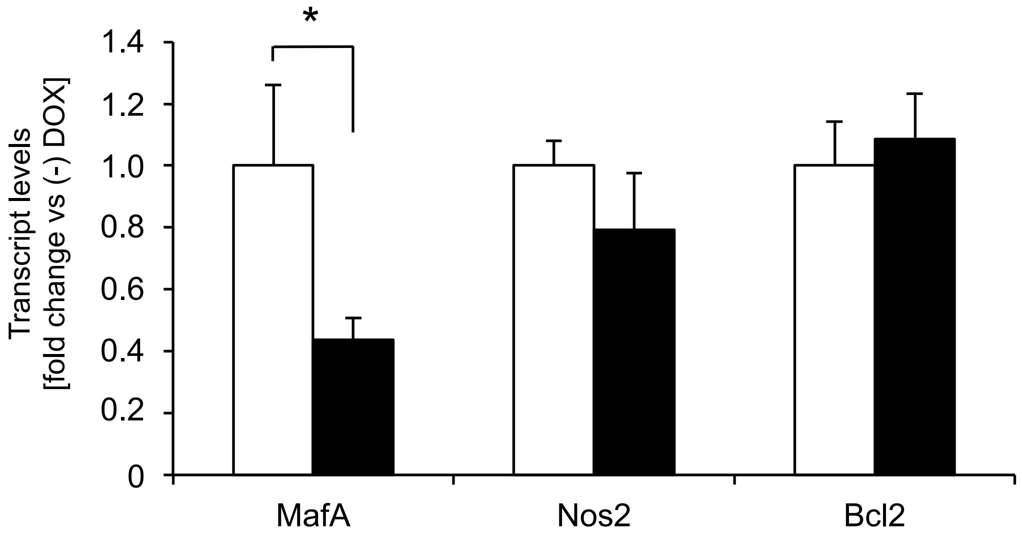

ESM Fig.3. Assessment of *Mafa*, *Nos2* and *Bcl2* transcript levels in PAX4 overexpressing islets. *Mafa*, *Nos2* and *Bcl2* transcript levels were measured in islets isolated from PAX4 transgenic mice treated (black bars) or not (white bars) with DOX for 1 month. Relative mRNA levels were normalized to the transcript levels of the housekeeping gene β-*actin*. Data are presented as fold change as compared to non-DOX treated mice. *n=4*, *p<0.05.
