# Supplementary material for: PAX4 preserves endoplasmic reticulum integrity preventing beta cell degeneration in a mouse model of type 1 diabetes mellitus
Source: Diabetologia. 2016 Jan 26;59:755–65. doi: 10.1007/s00125-016-3864-0 (PMC4779135; doi:10.1007/s00125-016-3864-0)
Supplement: Supplementary file 5 — (PDF 50 kb) [file 125_2016_3864_MOESM5_ESM.pdf]

ESM Table 1: Antibodies used in this study.

**Primary Antibody**	**Vendor**	**Cat. No.**	**Fold Dilution**
Mouse anti-insulin	Sigma	I2018-.2ML	500
Rabbit anti-glucagon	Cell Signalling	2760S	200
Mouse anti-glucagon	Sigma	G2654-.2ML	200
Rabbit anti-cleaved caspase-3	Cell Signaling	9661	150
Rabbit anti-Ki67	Thermo Scientific	RM-9106-SO	150
Mouse anti-53BP1	Santa Cruz Biotechnology	Sc-22760	500
**Secondary Antibody**	**Vendor**	**Cat. No.**	**Fold Dilution**
Goat anti-mouse IgG Alexa Fluor 568	Invitrogen	A11004	1000
Goat anti-rabbit IgG Alexa Fluor 488	Invitrogen	A11008	500
Donkey anti- goat IgG Alexa Fluor 488	Invitrogen	A11055	500
Donkey anti-mouse IgG Cy3	Jackson ImmunoResearch	715-175-151	800
