# Supplementary material for: PAX4 preserves endoplasmic reticulum integrity preventing beta cell degeneration in a mouse model of type 1 diabetes mellitus
Source: Diabetologia. 2016 Jan 26;59:755–65. doi: 10.1007/s00125-016-3864-0 (PMC4779135; doi:10.1007/s00125-016-3864-0)
Supplement: Supplementary file 6 — (PDF 43 kb) [file 125_2016_3864_MOESM6_ESM.pdf]

ESM Table 2: List of the 20 most highly up- and down-regulated genes (p-value < 0.05) in islets over-expressing Pax4 or Pax4R129W as sorted using the t-statistic algorithm.

Symbol	Pax4 versus Control	Symbol	Pax4R129W versus Control
Lgals9	6.43	4933402P03Rik	7.37
Pax4	6.38	Scn5a	5.93
Nnat	6.05	Olfr1045	5.84
Soat1	5.82	Ttll11	5.54
Nsdhl	5.39	Hapln3	5.44
Pdyn	5.29	Proca1	5.43
Rab8a	5.25	Nudt11	5.34
Cyb5b	5.11	Esyt3	5.31
Mrps21	5.09	Tmem200a	5.24
Rit2	4.98	Edn2	4.93
Rpl37	-4.12	Spry1	-4.90
Rps21	-4.15	Meg3	-4.99
Rps28	-4.27	Snapc3	-5.13
Inmt	-4.28	2610005L07Rik	-5.36
Snord8	-4.36	6820431F20Rik	-5.37
Snora16a	-4.45	6720401G13Rik	-5.39
Snord35b	-5.25	Ddx17	-5.87
4931414P19Rik	-5.40	Ankrd12	-6.16
Snora34	-5.64	Ddx26b	-6.31
Snord104	-8.10	Snora23	-6.33
