# Supplementary material for: PAX4 preserves endoplasmic reticulum integrity preventing beta cell degeneration in a mouse model of type 1 diabetes mellitus
Source: Diabetologia. 2016 Jan 26;59:755–65. doi: 10.1007/s00125-016-3864-0 (PMC4779135; doi:10.1007/s00125-016-3864-0)
Supplement: Supplementary file 7 — (PDF 62 kb) [file 125_2016_3864_MOESM7_ESM.pdf]

ESM Table 3: List of significant enriched KEGGs terms (adjusted p-value < 0.05) classified as up regulated in islets either overexpressing Pax4 or Pax4R129W.

Pax4 versus Control		Pax4R129W versus Control	
KEGG Term	-log10 (adjusted p-value)	KEGG Term	-log10 (adjusted p-value)
Protein processing in endoplasmic reticulum	8.32	Neuroactive ligand-receptor interaction	7.85
Lysosome	6.43	Cardiac muscle contraction	2.62
Steroid biosynthesis	6.43	Parkinson's disease	1.49
Cell cycle	5.93	Oxidative phosphorylation	1.42
Pyrimidine metabolism	4.38		
Glycosylphosphatidylinositol (GPI)-anchor biosynthesis	4.34		
B cell receptor signaling pathway	3.69		
Antigen processing and presentation	3.38		
Spliceosome	3.38		
Aminoacyl-tRNA biosynthesis	3.21		
DNA replication	2.29		
Cysteine and methionine metabolism	2.28		
Fc gamma R-mediated phagocytosis	2.27		
Hepatitis C	2.27		
Phagosome	2.27		
MAPK signaling pathway	2.27		
Glutathione metabolism	2.19		
RNA transport	2.08		
Biosynthesis of unsaturated fatty acids	2.08		
Glycosaminoglycan degradation	1.97		
Phenylalanine metabolism	1.85		
Thyroid cancer	1.85		
N-Glycan biosynthesis	1.85		
Leishmaniasis	1.76		
Fc epsilon RI signaling pathway	1.73		
Prion diseases	1.73		
One carbon pool by folate	1.71		
Insulin signaling pathway	1.68		
Endocytosis	1.66		
Glycerophospholipid metabolism	1.63		
Chronic myeloid leukemia	1.63		
Wnt signaling pathway	1.63		
Neurotrophin signaling pathway	1.61		
Purine metabolism	1.61		
Osteoclast differentiation	1.61		
Rheumatoid arthritis	1.56		
p53 signaling pathway	1.55		
Drug metabolism - other enzymes	1.50		
Toxoplasmosis	1.47		
Proteasome	1.43		
Glycolysis / Gluconeogenesis	1.43		
Pentose phosphate pathway	1.42		
Jak-STAT signaling pathway	1.42		
Mismatch repair	1.36		
Citrate cycle (TCA cycle)	1.36		
Nucleotide excision repair	1.35		
Acute myeloid leukemia	1.35		
