# Supplementary material for: PAX4 preserves endoplasmic reticulum integrity preventing beta cell degeneration in a mouse model of type 1 diabetes mellitus
Source: Diabetologia. 2016 Jan 26;59:755–65. doi: 10.1007/s00125-016-3864-0 (PMC4779135; doi:10.1007/s00125-016-3864-0)
Supplement: Supplementary file 8 — (PDF 51 kb) [file 125_2016_3864_MOESM8_ESM.pdf]

ESM Table 4: List of significant enriched KEGGs terms (adjusted p-value < 0.05) classified as down regulated in islets overexpressing Pax4 or Pax4R129W.

Pax4 versus Control		Pax4R129W versus Control	
KEGG Term	-log10 (adjusted p-value)	KEGG Term	-log10 (adjusted p-value)
Ribosome	28.73	Ubiquitin mediated proteolysis	7.88
Cardiac muscle contraction	1.73	Spliceosome	7.80
		RNA transport	4.40
		Protein processing in endoplasmic reticulum	3.74
		Ribosome biogenesis in eukaryotes	3.74
		Aminoacyl-tRNA biosynthesis	3.00
		mRNA surveillance pathway	2.89
		Prostate cancer	2.62
		Phosphatidylinositol signaling system	2.62
		Oocyte meiosis	2.62
		Inositol phosphate metabolism	2.33
		Chagas disease (American trypanosomiasis)	2.20
		Endocytosis	1.90
		Lysosome	1.77
		NOD-like receptor signaling pathway	1.77
		Progesterone-mediated oocyte maturation	1.75
		Cell cycle	1.75
		Bacterial invasion of epithelial cells	1.75
		Pancreatic cancer	1.50
